# The Coax Dipole: A fully flexible coaxial cable dipole antenna with flattened current distribution for body imaging at 7 Tesla

**DOI:** 10.1002/mrm.28983

**Published:** 2021-08-19

**Authors:** Carel C. van Leeuwen, Bart R. Steensma, Dennis W. J. Klomp, Cornelis A. T. van den Berg, Alexander J. E. Raaijmakers

**Affiliations:** ^1^ Department of Radiology University Medical Center Utrecht Utrecht The Netherlands; ^2^ Biomedical Engineering Department Eindhoven University of Technology Eindhoven The Netherlands

**Keywords:** body imaging, coaxial, dipole, flexible, RF‐transmit, ultra high field

## Abstract

**Purpose:**

The *coax dipole antenna*, a flexible antenna for body imaging at 7T is presented. Similar to the high impedance coil, this coaxial cable antenna is fed on the central conductor and through gaps in the shield, the current passes to the outside of the antenna to generate B_1_ field. This could achieve more favorable current distributions and better adaptation to the body curvature.

**Methods:**

Finite difference time domain (FDTD) simulations are performed to optimize the positions of the gaps in the shield for a flat current profile. Lumped inductors are added to each end to reduce losses. The performance of a single antenna is compared to a fractionated dipole using B_1_ maps and MR thermometry. Finally, an array of eight coax dipoles is evaluated in simulations and used for in‐vivo scanning.

**Results:**

An optimal configuration is found with gaps located at 10 cm from the center and inductor values of 28 nH. In comparison to the fractionated dipole antenna, in single antenna phantom measurements the coax dipole achieves similar B_1_ amplitude with 18% lower peak temperature. In simulations, the eight‐channel array of coax dipoles improved B1 homogeneity by 18%, along with small improvements in transmit efficiency and specific absorption rate (SAR). MRI measurements on three volunteers show more consistent performance for the coax dipoles.

**Conclusion:**

The coax dipole is a novel antenna design with a flattened current distribution resulting in beneficial properties. Also, the flexible design of the coax dipoles allows better adaptation to the body curvature and can potentially be used for a wide range of imaging targets.

## 1 INTRODUCTION

The development of an optimal multi‐transmit coil for ultra‐high field (B_0_ ≥ 7T) imaging has been the subject of on‐going scientific investigation. Especially for ultra‐high field MRI of the body, the commonly used birdcage coil cannot be used because of strongly reduced transmit efficiency and homogeneity. Instead, dipole antennas are commonly used as transmit or transmit/receive elements for ultra‐high field MRI.^1^ Different strategies can be used to optimize the dipole sensitivity for body^2^ and brain imaging^3^, ^4^ or to decrease peak local specific absorption rate (SAR).^5^, ^6^, ^7^, ^8^, ^9^, ^10^ Dipole antennas can be favored over loop coils for ultra‐high field imaging since dipole antennas perform better for deeply situated imaging targets.^11^ From a theoretical point of view, antennas with z‐oriented current distributions (dipoles or meander strip line antennas^12^) appear to become more beneficial for increasing frequencies at higher field strength.^13^ Since dipole antennas are inherently decoupled from loop coils^14^, ^15^, ^16^ loops and dipoles can also be combined together in multi‐transmit arrays to further improve sensitivity.

The self‐resonance length of a plain dipole antenna loaded by human tissue at 7T is roughly 38 cm. However, dipole antennas used for MR are generally shorter. A length of 30 cm is better aligned to the size of most imaging targets, resulting in a more efficient antenna.^2^, ^11^ However, the current distribution on these short antennas has a triangular shape, with a maximum at the source. In theory, an antenna with a flat current distribution will perform better: A more homogeneous current distribution results in more homogeneous fields along the length of the antenna, yielding better B_1_ coverage and lower peak SAR values. At the self‐resonance length, the current profile has a sinusoidal shape. Many dipole designs that have lower peak SAR compared to plain dipoles^2^, ^5^, ^7^, ^8^ achieve flatter current profiles by increasing the electrical length of the antenna, while maintaining a compact form.

In addition to a good electromagnetic performance, more practical aspects of the coil design should be considered when developing new coil arrays. A lightweight and flexible coil design improves ergonomics for the subject, makes for easier handling of the coil and can improve coil loading in various circumstances, which in turn improves electromagnetic performance.

Recently, the use of shielded coaxial loop coils has been demonstrated to achieve improved decoupling,^17^, ^18^, ^19^ lower peak SAR^20^ and a flexible coil design which allows for better coil loading compared to rigid loop coils.^21^ In this work, we investigate if and how we can translate the concept of coaxial loop coils to dipole antennas and evaluate whether it improves the overall performance. We investigated through simulations how to tune and optimize the design of the coaxial dipole to achieve a flat current distribution on the outer shield of the coaxial cable. Additional inductors were needed at the dipole endings to reduce losses and to facilitate matching of the antenna to 50 ohm. We demonstrate in simulations and experiments that our novel design achieves lower peak SAR while maintaining the same transmit efficiency as a fractionated dipole antenna. By using a flexible antenna design, it is possible to achieve a constant distance to the body under a wide range of circumstances, and a very light‐weight coil array design can be achieved, which improves patient comfort.

## 2 METHODS

Analogous to the coaxial cable loop coil, this study presents a dipole antenna constructed from coaxial cable named the *coax dipole antenna*. First, we outline the design process of the antenna, through a series of FDTD simulations. Second, we show how the antennas are constructed and the performance of a single antenna is tested on a homogeneous phantom. Finally an array of eight coax dipoles is evaluated for body imaging at 7T. FDTD simulations are used to evaluate the performance in terms of B_1_ and SAR, using a realistic human model. In‐vivo, the performance is evaluated both quantitatively, by measuring the B_1_ efficiency for prostate imaging in a number of volunteers, and qualitatively, by cardiac images where the expected benefit of flexible antennas, adjusting their shape to the chest curvature, is more apparent. An array of fractionated dipole antennas^2^ is used for comparison, where possible.

### 2.1 Simulation‐based design of the coax dipole

**FIGURE 1:**
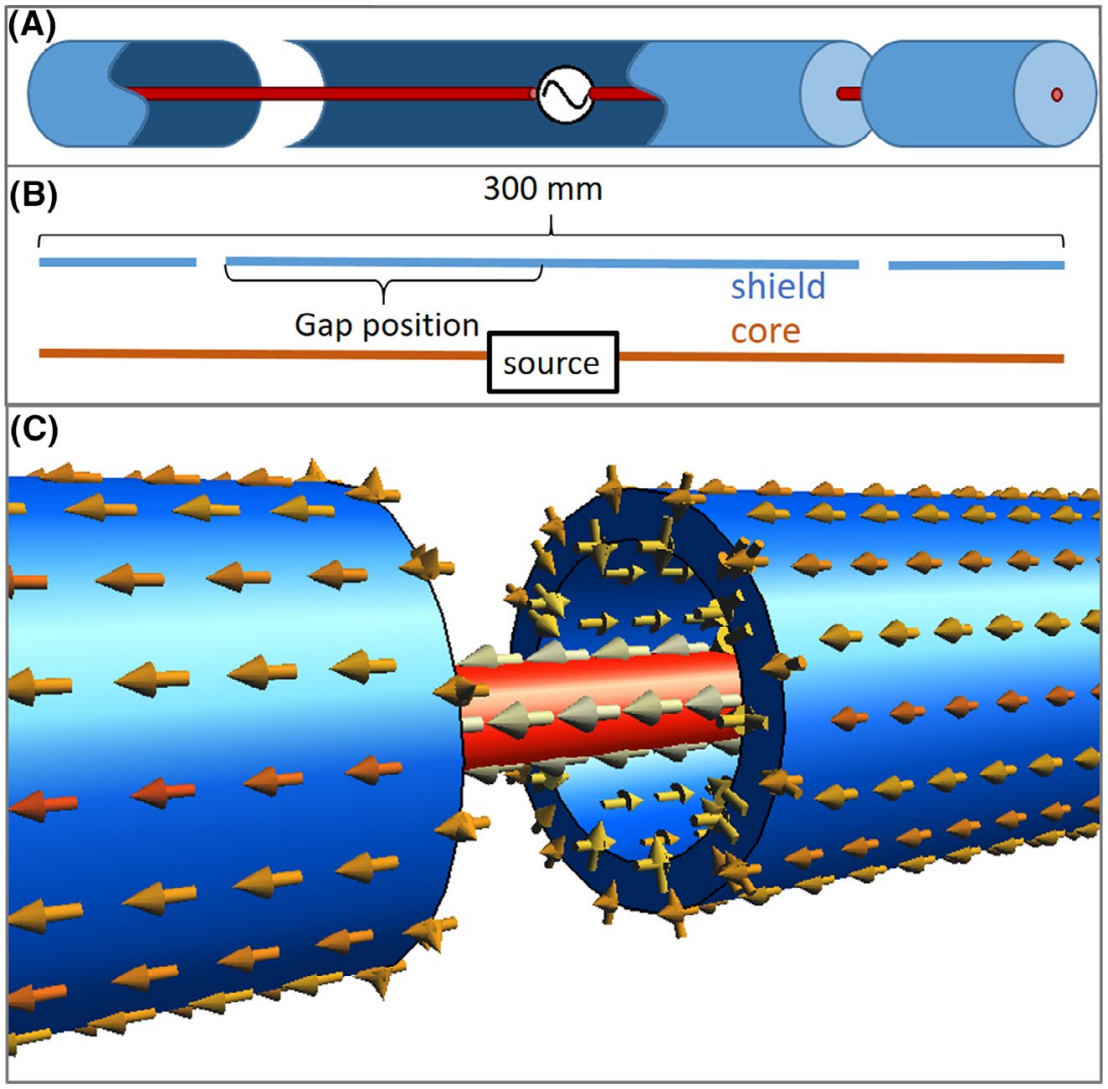
A, Schematic drawing of a coax dipole antenna: the source is connected to the core (central conductor) of a coaxial cable. The shield (outer conductor) is interrupted by gaps. B, Diagram of antenna layout. Position of the gap is indicated by its distance from the center. C, Close‐up image of gap in the shield. Surface currents (indicated by arrows) show how the gap allows current to flow on the outside of the shield

An antenna made entirely of coaxial cables, driven on the shield of the cables, is effectively just a plain dipole antenna with peak current and high SAR values in the center. When driven on the core (central conductor) of the cable, it is shielded and does not radiate. However, if in this case the shield (outer conductor) is interrupted by a gap, current will flow onto the outside of the shield and the antenna becomes radiative. Figure 1 schematically shows an antenna made of coax cables, with gaps in the shield. Figure 1C depicts how the gap causes current to flow on the outside of the shield. The first step in our design process was to determine how the current distribution that results in radiation depends on the position of the gap in the shield. A series of finite difference time domain (FDTD) simulations (Sim4Life, Zurich Medtech, Switzerland) was performed, modeling the behavior of a 30 cm long coax dipole antenna. A source was connected to the core in the center of the antenna. On each arm of the dipole, the shield of the cable was interrupted by a 3 mm wide gap. (Figure 1A,B) The distance between the source and the gap was varied from 25 mm to 125 mm. Additionally, a simulation was performed with only a gap in the center, where the source was connected directly to the shield of the cable (ie, a plain dipole). In each case the antenna was positioned at 2 cm from a homogeneous phantom (σ = 0.5 S/m, ε_r_ = 46). The coaxial cable was modeled after a commercially available cable (Huber Suhner RG223u) with the core and shield modeled as Perfect Electrical Conductor (PEC). The antenna was voxelized at a resolution of 1.5 mm along its length and 0.16 mm along its cross‐section, requiring 32 × 32 grid lines along its cross‐sectional area and a total number of cells of 3.8 million. Performance of the antennas was evaluated in terms of the B_1_ field strength at 10 cm depth inside the phantom and maximum SAR in the phantom. Additionally, current distributions on the core and shield were determined by numerically integrating the Maxwell–Ampère equation around each conductor. These currents are used to estimate the metal losses in each conductor, using a conductivity of 5.9 × 10^7^ S/m and skin depth of 3.8 µm.

**FIGURE 2:**
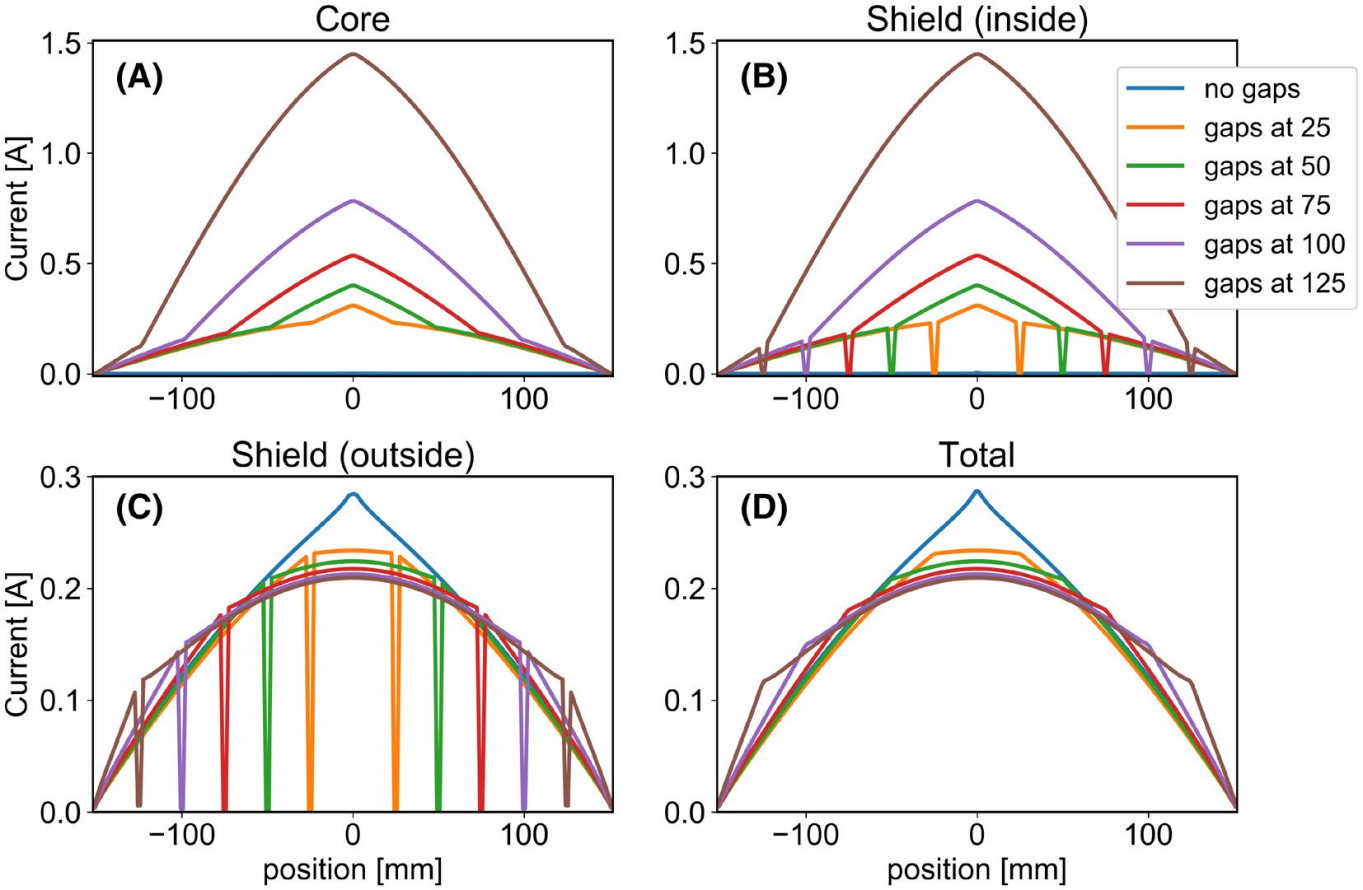
Magnitude of complex current distributions (computed by numerically integrating the Maxwell‐Ampère equation) on each layer of the antenna, for coax dipoles with gap positions as indicated (in mm). Total current refers to the complex sum of current distributions of the layers. All results are normalized to 1W accepted power

**FIGURE 3:**
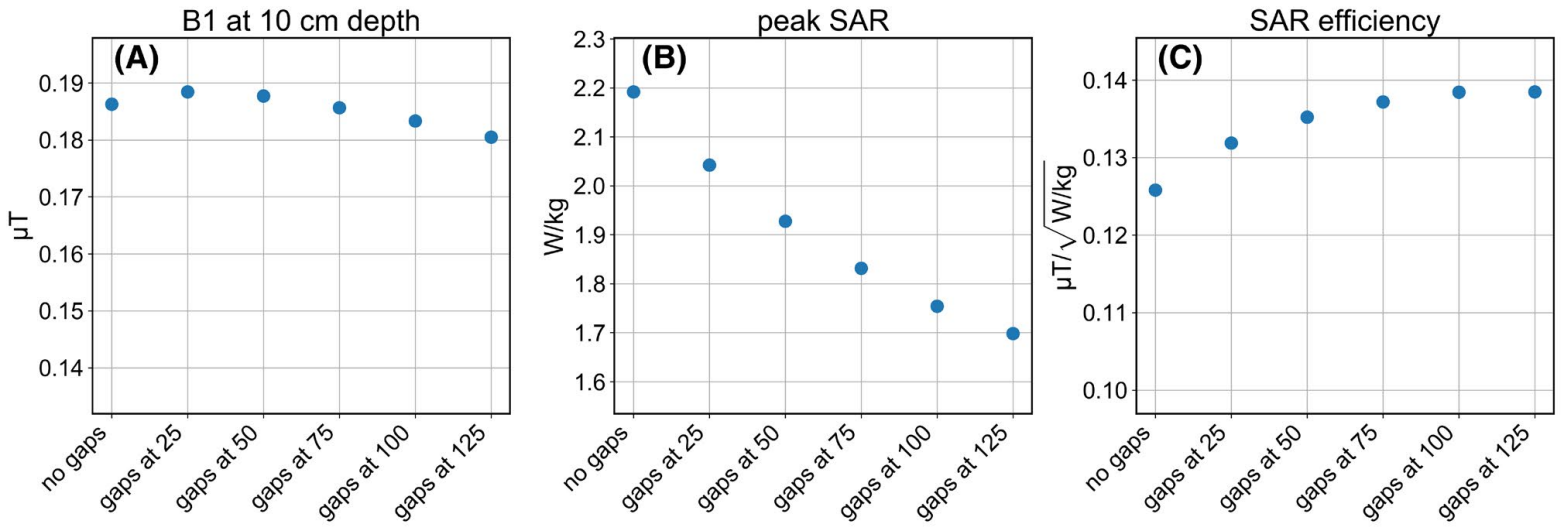
Metrics of interest for coax dipoles with gaps at various positions (in mm). A, B_1_ amplitude under the center of the antenna, at 10 cm depth in the phantom. B, Peak SAR value over the phantom. C, SAR efficiency: B_1_ at 10 cm depth divided by square root of peak SAR. All results are normalized to 1 W accepted power

**FIGURE 4:**
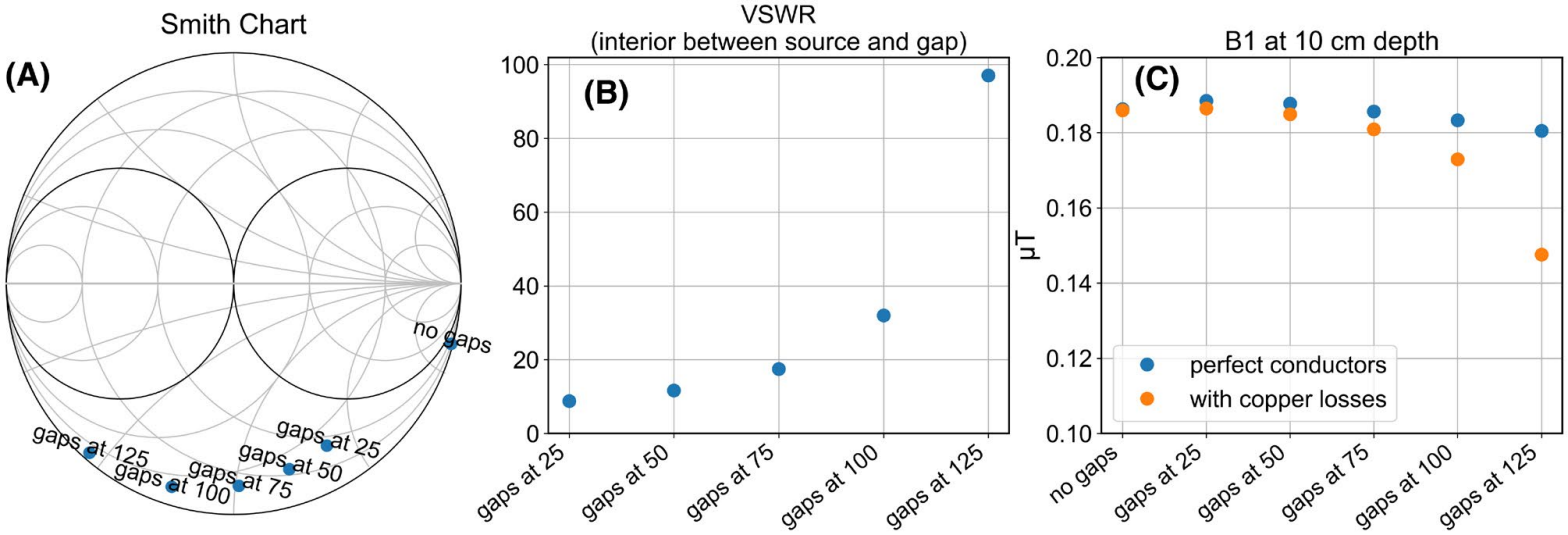
Downsides of coax dipoles with gaps far from the center. A, Reflection coefficients of each antenna displayed in Smith chart. B, VSWR on central conductor of the antenna, on the section between the source and gap. C, Same as Figure 3A, but now with copper losses included

**FIGURE 5:**
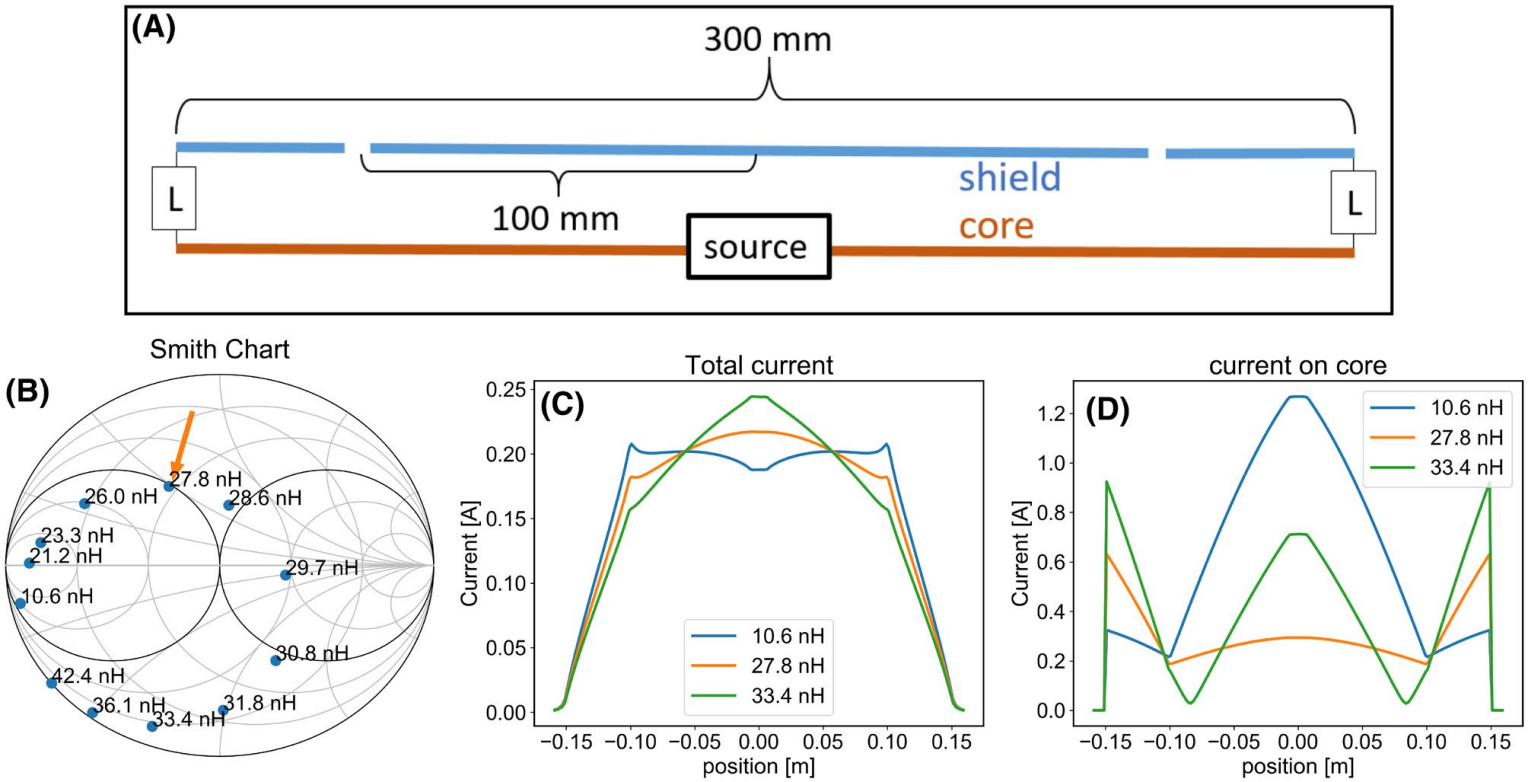
Proposed improvement of coax dipole antenna (with gaps at 100 mm from center) by adding inductors at both ends of the dipole. A, Diagram of antenna layout, with inductors added. B, Reflection coefficient measured at the source, for various inductance values. total current on the antenna (C) and current on antenna core (D) for three inductance values. The value of 27.8 nH (indicated by the arrow in B) largely solves the problems (shown in Figure 4) that occurred in the antenna without inductors

As results will show (Figure 2), the antennas with gaps furthest from the center resulted in the flattest current distributions, yielding the best SAR efficiency (Figure 3). However, these antennas suffer from two critical related downsides: A very high current amplitude on the core conductor, and a very low real part of the impedance measured at the port, which complicates impedance matching (Figure 4). To solve these problems, a lumped element connecting the core to the shield was added to each end of the antenna. This lumped element can be used to effectively match the impedance transition that propagating waves from the source along the coaxial cable encounter when they hit on the gap. Using various lumped element values, we explored the possibility of alleviating the aforementioned downsides, while maintaining a beneficial current distribution on the outside of the shield. Each antenna was simulated using FDTD as previously, but with the extra lumped element added. Network co‐simulation^22^, ^23^ was used to attempt a wide range of lumped element values, without requiring a new simulation for each value. Antenna performance was evaluated not only by determining transmit‐ or SAR‐efficiency, but also by carefully observing how the current amplitude on the core and impedance measured at the port behave as a function of lumped element value (Figure 5).

### 2.2 Antenna construction

**FIGURE 6:**
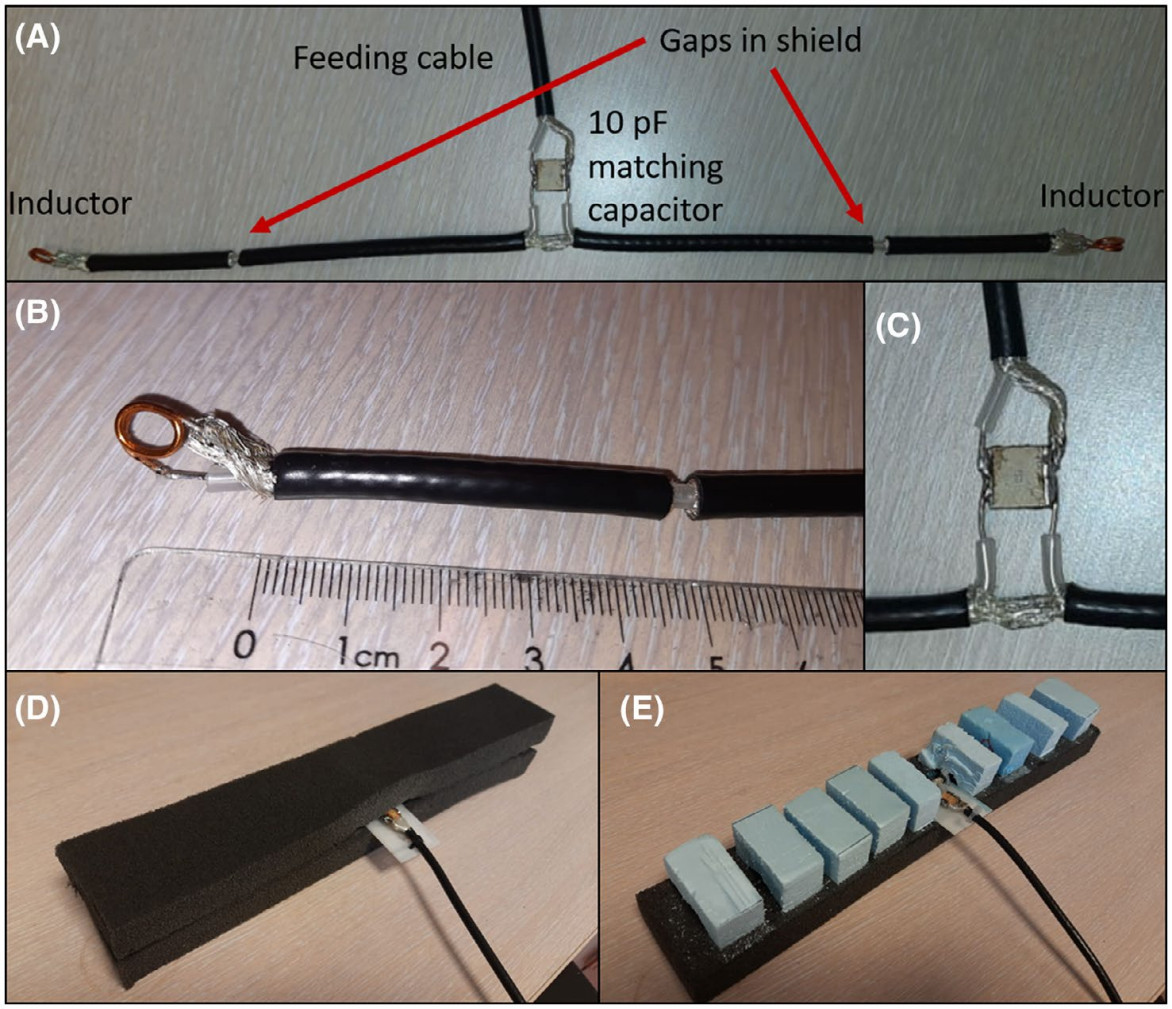
Photographs of the coax dipole with inductors. A, top view of the antenna, with various components as indicated. B, close‐up view of one end of the antenna, showing the inductor and gap. C, close‐up view of matching circuit containing just one parallel capacitor. Photographs of A, B, and C were taken before addition of foam housing. D, Anterior antenna elements with foam housing added. E, Posterior elements with small sections of rigid foam, to ensure proper distance between the body and antenna as the posterior elements carry the weight of the subject in the scanner

The coax dipole antenna is made from two sections of coaxial cable (Huber Suhner RG223u, characteristic impedance: 50 Ω). On each section, two centimeters of insulation layer is removed, and the core is brought outside of the shield to enable connection to a feeding cable and matching capacitor. The two antenna sections are joined by soldering the shields together, creating a single antenna. The two bits of core of each section, protruding from the antenna, constitute the feeding point (Figure 6C). In both sections, a 3 mm wide gap was made in the shield. The gaps are placed 20 cm apart, placed symmetrically with the feeding point in between them. On each end of the antenna, the core and the shield of the coaxial cable are connected by an inductor. Inductors are hand wound using 38 mm long sections of annealed copper wire (thickness 1.5 mm). The inductance value is determined by measuring the reflection at the feed port and computing the admittance, *Y*. The correct inductance value will result in Re(*Y*) = 0.02 S, corresponding to the location in the Smith chart indicated by the arrow in Figure 5B. The antenna is matched by adding a parallel capacitor (American Technical Ceramics, 800E series, 10 pF) to the feeding point. Finally strain relief is added, and the antenna is placed between two 2.5 cm thick layers of flexible foam (see Figure 6D). For four posterior antennas (which carry significant weight), the flexible foam on one side was replaced with small sections of rigid foam (see Figure 6E). This was done to maintain a flexible housing while maintaining at least 2 cm distance between the body and conducting parts of the antenna. As in previous work by our group,^2^ no baluns or cable traps were used for the transmit antennas. The extensive experience with these antennas suggests that baluns are not needed, possibly due to the heavy loading of the antenna. Possibly, small improvements in efficiency can be achieved for the coax dipole array by using baluns. But since our reference array of fractionated dipoles does not use them, for a fair comparison, we took the same approach with the coax dipoles. Common mode currents are reduced by routing the cables perpendicularly away from the dipoles and as an additional safety measure the cables are always kept more than 2 cm away from the subject.

### 2.3 Single‐channel phantom measurements

Antenna performance was measured on a 7T MRI scanner (Philips Achieva, Philips Healthcare, Best, the Netherlands) using a body phantom (σ = 0.5 S/m, ε_r_ = 46^24^) and a single coax dipole antenna. Transmit efficiency was assessed by measuring B_1_ amplitude maps (method: DREAM.^25^ FA/steFA/TE/TR: 10°/60°/1.4/4 ms). SAR was assessed by MR Thermometry using the proton resonance frequency shift method^26^ (FA/TE/TR: 11°/10/15 ms). Heating was produced by 100 kHz off‐resonance block pulses 20 W average power, over 3 min. Both measurements were repeated with a fractionated dipole antenna^2^ for comparison.

### 2.4 Eight‐channel FDTD simulations ‐ simulation setup

Eight coax dipoles were positioned for cardiac imaging around a realistic human model (*Duke*, ITIS foundation^27^). The 30 cm fractionated dipole antennas, used for comparison, included a 2 cm thick Plexiglas spacer and were positioned as tightly as possible around the same model. The coax dipoles were positioned by starting from the same locations as the fractionated dipoles and moving them toward the body. As allowed by their flexible structure, the coax dipoles were bent and tilted to adjust to the body contour such that a distance of 2 cm between the body and any conducting component is maintained over the entire length of the antenna (see Figure 9A). Two medial anterior elements of the fractionated dipoles had a 21° angle between the two dipole sections, which allows them to better adjust to the contours of the body^28^ (see Figure 9F). Each antenna contained additional matching lumped elements at the port, and the coax dipoles contained lumped inductors at the end, but all these lumped elements were simulated as ports for circuit co‐simulations. The body model was voxelized at a resolution of 3 mm isotropic or smaller. (locally down to 0.5 mm in x and y directions, which was required to accurately model the coax dipoles.) The coax dipole antennas were voxelized on subgrids, with a resolution of 1.0 mm along its length and 0.17 to 0.25 mm along the cross sectional area. The total number of cells was 14.2 million for the fractionated dipole antennas, and 32.4 million for the coax dipoles.

#### 2.4.1 Eight‐channel FDTD simulations ‐ data analysis

Network co‐simulation^22^, ^23^ was used to combine the fields of the different ports in each antenna, while replacing some ports with lumped element values to tune and match the antennas. Initially, the inductance value of the lumped element at each end of the coax dipoles was determined by computing the admittance *Y* at the source and choosing the inductance that resulted in Re(*Y*) = 0.02 S. Additionally, after visual inspection of the transmit field distributions for single antennas, the inductance values were tweaked slightly (values ranged from 18 to 27 nH) to evenly distribute the current over both arms of the antenna (while maintaining the same source impedance), correcting for asymmetries caused by staircasing effects, which arose from the fact that in some antennas the arms were oriented differently with respect to the rectilinear FDTD grid. The matching lumped elements at the source were optimized to minimize reflections.

The eight channels were combined by numerically optimizing their phases ϑ:

(1) $$\vartheta =\arg_{\vartheta }\min\left\{{\text{SD}}_{\left|B_{1}^{+}\right|}\left(\mathrm{ROI}\right)+\lambda P_{\text{deposited}}\right\}$$

where ${\text{SD}}_{\left|B_{1}^{+}\right|}\left(\mathrm{ROI}\right)$ refers to the standard deviation of the $B_{1}^{+}$ distribution in a target region of interest (ROI) containing the whole heart, P_deposited_ refers to the total deposited power and λ is a regularization parameter which was given a value of 0.01. All channels were given the same amplitude. The SAR distribution was averaged over 10 gram mass cubes to obtain SAR_10g_. The performance of each array is assessed in terms of B_1_ amplitude (average B_1_ over the ROI), B_1_ homogeneity (*coefficient of variation* over the ROI), inter‐element coupling, deposited power and peak SAR_10g_.

### 2.5 Eight‐channel in vivo measurements

After obtaining institutional review board approval, three healthy volunteers (body mass index [BMI] 27.1, 20.3, 25.2; age 30, 30, 41) were scanned at a 7T scanner (Philips Achieva, Philips Healthcare, Best, the Netherlands) using an array of eight coax dipole antennas and eight fractionated dipoles for reference. To ensure the local SAR limits are not exceeded, an average input power limit of 4W per channel was used, which is the same as for the fractionated dipole antennas and which includes safety factors for inter‐subject variation^29^ and modeling and power measurement uncertainty. In all scans B_1_ phase shimming was performed by measuring the average transmit phase in a target region using single‐channel gradient echo images and optimizing the transmit phase of every channel, such that the phases of the single‐channel gradient echoes interfere constructively in the target region.^30^ All eight elements were used for both transmission and reception, no additional receiving elements were used. The same set of transmit/receive (T/R) switches was used for both arrays. B_1_ amplitude maps (AFI‐method,^31^ FoV: 263 × 433 × 30 mm^3^, resolution: 3.9 × 3.8 × 10 mm^3^, TE/TR1/TR2/FA: 2.2 ms/50 ms/250 ms/65°) were obtained of the prostate of three healthy volunteers, using coax dipoles and fractionated dipoles for comparison. Each volunteer was scanned twice using two separate sessions, on separate days.

Additionally, cardiac cine scans (resolution: 1.3 × 1.3 × 8 mm^3^, 30 frames, TE/TR/FA: 3.2 ms/6.6 ms/15°, scan duration: 10 s) were performed on one volunteer using the coax dipoles, to demonstrate the capability of these antennas to obtain good coverage over the heart.

## 3 RESULTS

### 3.1 Simulation‐based design of the coax dipole

Figure 2 shows the simulated amplitude of the current on each layer of conductor as well as the total current, for different gap positions. Results for a dipole without gaps (except for one in the center, where the source is connected to the shield) are also included. In this case no current runs on the inside of the coaxial line. A flatter total current distribution is produced when placing the gaps further away from the center. Figure 3 shows how the $B_{1}^{+}$ and SAR levels depend on gap position. We see that with increasing gap distance the $B_{1}^{+}$ is reduced only slightly and peak SAR value becomes significantly lower. The antennas with the flattest current distributions (gaps at 100 and 125 mm) yield the best SAR efficiency. However, Figure 2B,C show that these antennas have a very high current amplitude on the core and inside of the shield.

Figure 4 outlines how this strong current causes these antennas to perform poorly in practice. Figure 4A shows a Smith chart with the reflections measured at the sources of the antennas. The source impedances of the antennas with gaps at 100 and 125 mm have a very low real component. To match these antennas, a parallel lumped element with a very low absolute impedance is required. Figure 4B shows the voltage standing wave ratio (VSWR) of the wave traveling from the source to the gap along the waveguide constituted by the interior of the antenna. A high VSWR indicates a large part of the energy is reflected at the gap, and only a small portion of the energy is transferred to the outside of the shield. Figure 4C shows how the results of Figure 3A change if copper losses are accounted for: The strong currents on the core cause a significant reduction in $B_{1}^{+}$ field strength.

Our final design, shown schematically in Figure 5A, solves these aforementioned problems. The gap is positioned at 100 mm from the source and two lumped inductors are added to the ends of the antenna, connecting the core to the shield. Figures 5B‐D show how a value of 27.8 nH results in a combination of beneficial properties: The antenna can easily be matched using a single parallel capacitor, as shown by the Smith chart of Figure 5B. The total current distribution (Figure 5C) is relatively flat over the central section of the antenna and the current amplitude on the core (Figure 5D) is low. Comparing the final design to the version without inductors (but the same gaps at 100 mm), the B1 amplitude and SAR efficiency are the same, but the copper losses are halved and the antenna is much easier to match to 50 Ohm. Figure 6A‐C show photographs of the constructed coax dipole antenna, before addition of the foam housing and strain relief. Figure 6D,E show photographs with foam housing and strain relief included.

### 3.2 Single‐channel phantom measurements

**FIGURE 7:**
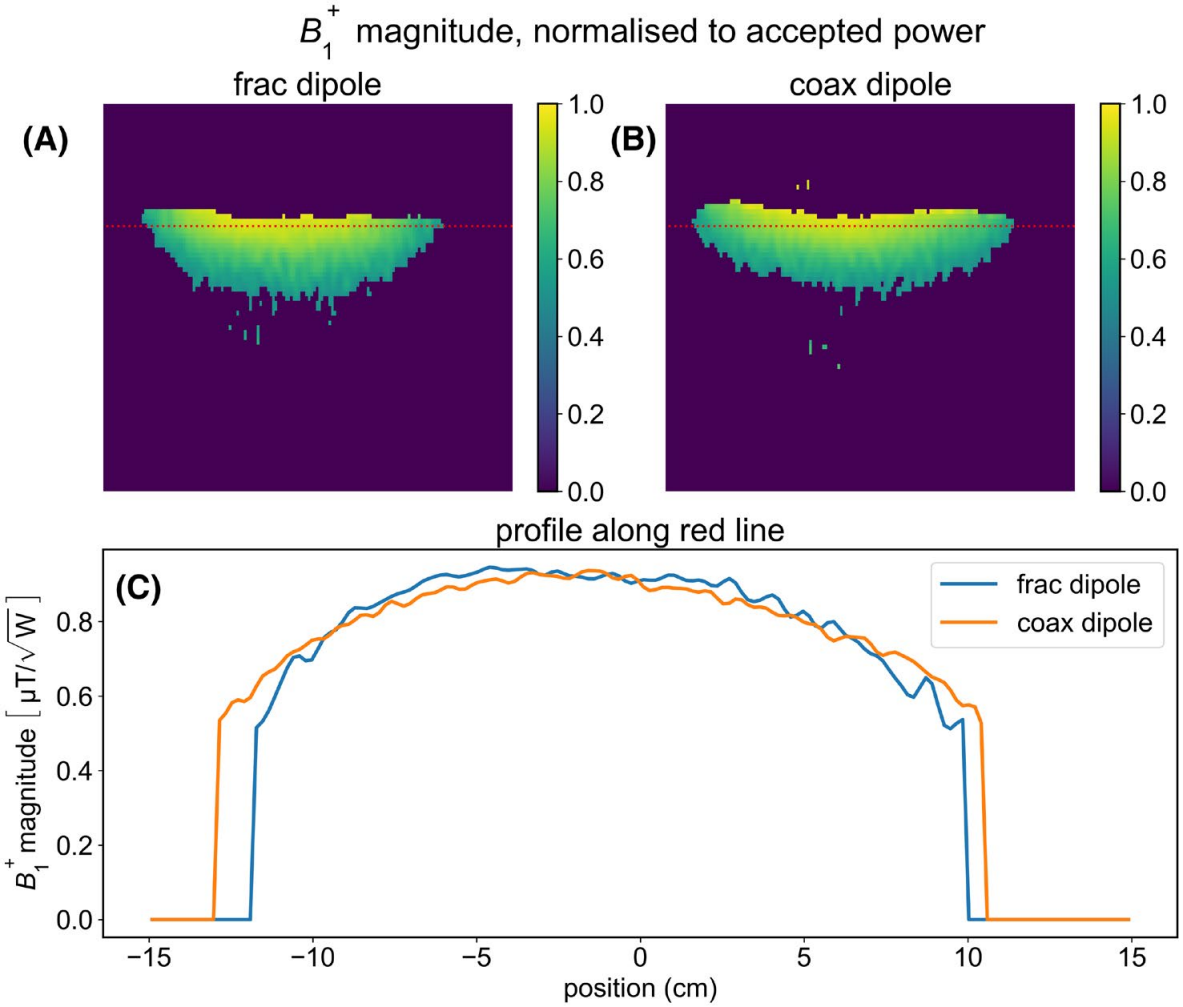
Single‐channel B_1_ comparison on a homogeneous phantom. A, Sagittal B_1_ map obtained with fractionated dipole. B, Sagittal B_1_ map obtained with coax dipole. C, Profile of B_1_ amplitude directly under antenna. Position is indicated by dotted red line in figures A and B

**FIGURE 8:**
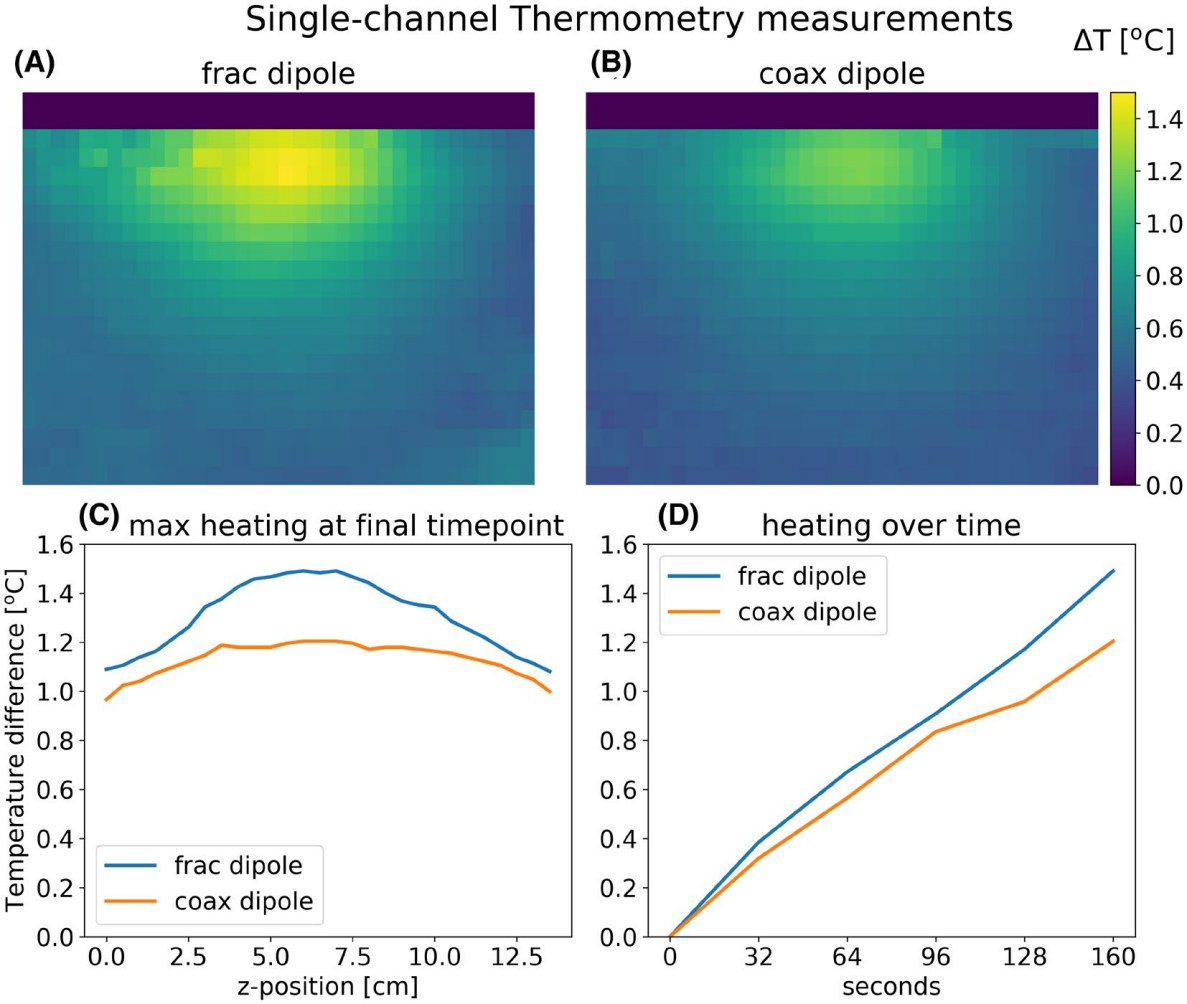
Single‐channel MR thermometry measurements on a homogeneous phantom. A,B, Maximum intensity projections of temperature maps onto the transverse plane. C, Maximum heating at the final timepoint, in each transverse slice. D, maximum heating over the whole volume for each timepoint

Figures 7 and 8 show results of the single‐channel phantom measurements. In Figure 7, we see the coax dipole produces roughly the same B_1_ amplitude as the fractionated dipole: its amplitude is slightly lower in the center and slightly higher toward the edges but the differences are within a few percent. However, in Figure 8, we see that the coax dipole causes 18% less peak heating.

### 3.3 Eight‐channel FDTD simulations

**FIGURE 9:**
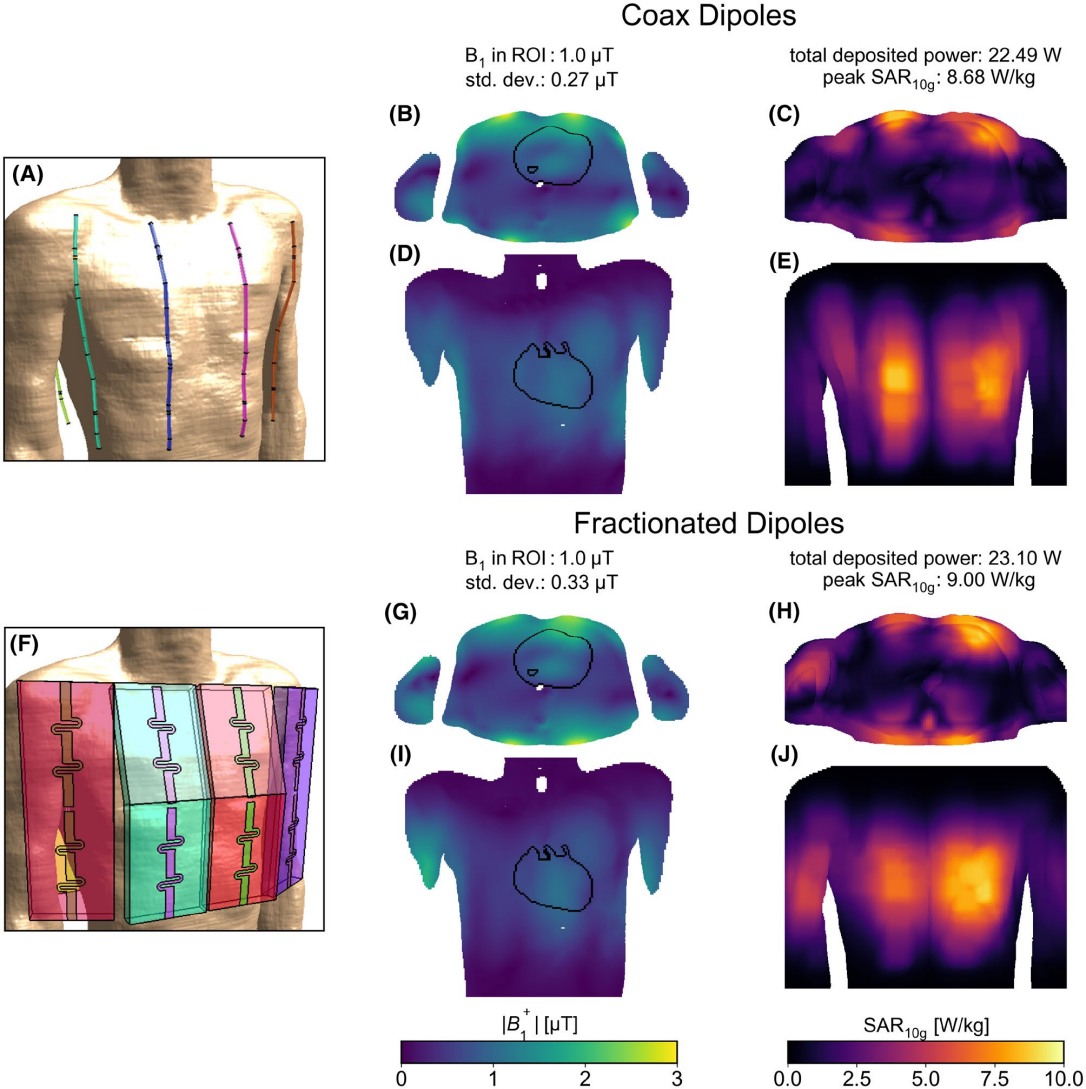
Eight‐channel simulation results. A,F, screenshots of the body model with antennas. (B,D,G,I) Transverse and coronal slices of B_1_ distributions, through the middle of the ROI (outlined in black) containing the whole heart. C,E,H,J, Maximum intensity projections of the 10‐gram averaged SAR distribution onto the transverse and coronal planes. All field distributions are scaled to an average of 1 μT in the ROI, for easy comparison of the other metrics (summarized in the figure)

The results of the eight‐channel FDTD simulations are shown in Figure 9. The field distributions are normalised to an average B_1_ amplitude of 1μT in the target region. The coax dipoles perform better in terms of homogeneity: the standard deviation of the B_1_ amplitude is reduced by 18%. In terms of transmit efficiency and SAR efficiency, the improvements are more modest: total deposited power is reduced by 2.6% and peak SAR_10g_ is reduced by 3.6%. The simulated inter‐element coupling is slightly higher for the coax dipoles: The strongest nearest‐neighbor coupling observed was −10.5 dB, with an average of −16.5 dB. With the fractionated dipoles the strongest and average nearest‐neighbor coupling were −14.2 dB and −16.9 dB, respectively.

### 3.4 Eight‐channel in vivo measurements

**FIGURE 10:**
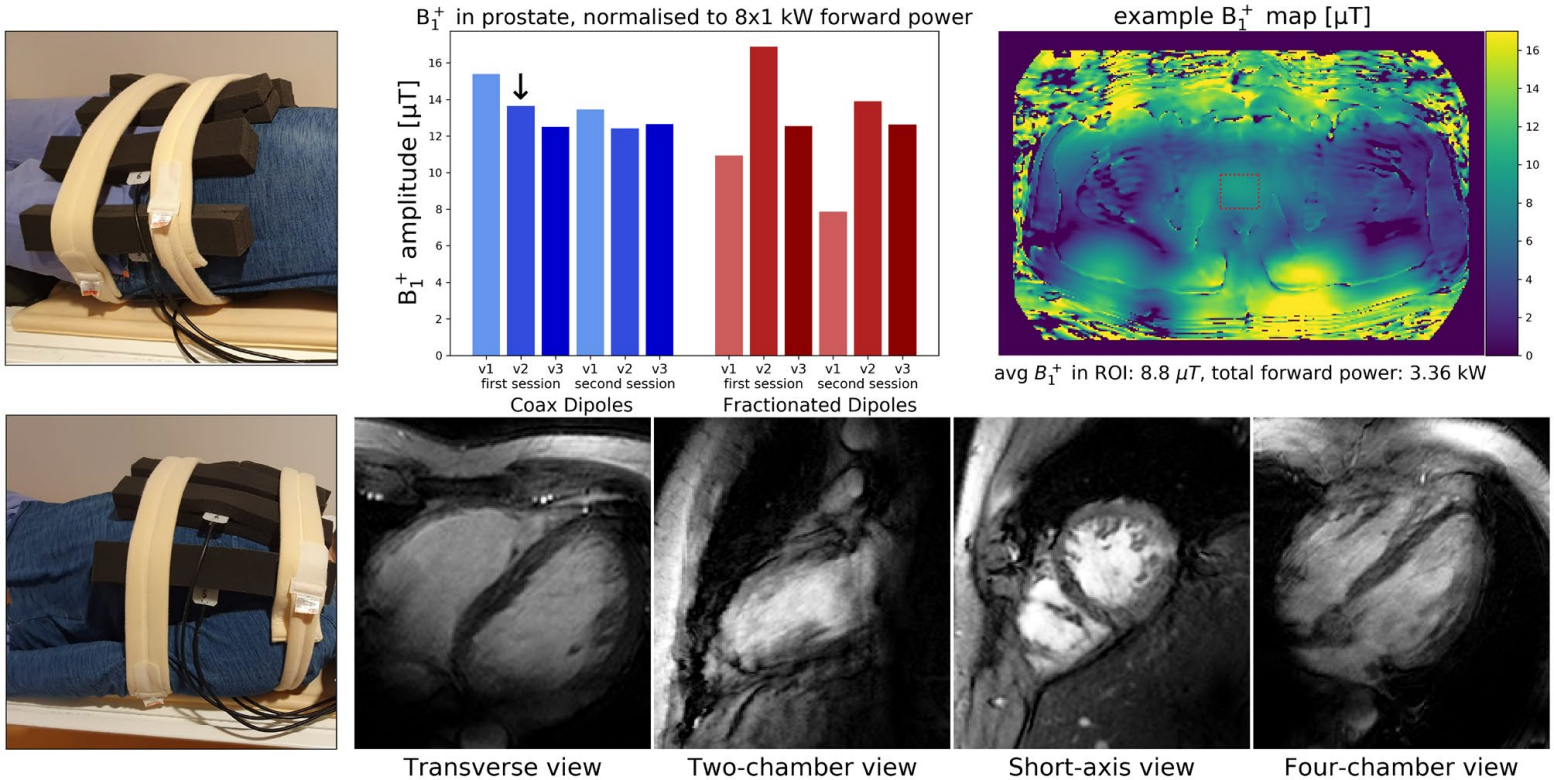
In vivo imaging results. Top row shows prostate imaging results. A, Photograph of antenna array on a volunteer, positioned for prostate imaging. B, Average B_1_ levels in the prostate, normalized to 8 × 1 kW forward power. Three volunteers were scanned twice with each array. C, Example B_1_ map of the volunteer indicated by the arrow in subfigure B. An average of 8.8 μT was obtained in the prostate with 3361 W forward power. Bottom row shows cardiac imaging results. D, Photograph of antenna array on a volunteer, positioned for cardiac imaging. E‐H, Single frames of cardiac cine images, in various views as indicated. Movies of the whole cardiac cycle are available as supplementary material

The results of the in‐vivo scans are shown in Figure 10. Figure 10B shows the B_1_ amplitude measured in the prostate of three volunteers, in two separate sessions. The average B_1_ amplitude achieved by the coax dipoles seems to be similar to the fractionated dipoles, but the coax dipoles show more consistent performance over the different volunteers. The highest and lowest values are both achieved by the fractionated dipoles. Figure 10C shows an example B_1_ map of the scan indicated by the arrow in Figure 10B. In this case an amplitude of 8.8μT in the prostate was achieved with 3361 W of forward power. The bottom row (Figure 10E‐H) shows one frame of cardiac cine scans, in various views. Movies of the entire cardiac cycle can be found in the Supporting Information Material, which is available online.

## 4 DISCUSSION

This work has presented a new design for a flexible dipole antenna made from coax cables, operating at 7T. It was inspired by recent developments of loop coils made from coax cables,^17^, ^18^, ^21^ and builds on the same principles: A wave propagates on the inside of the cable and by making gaps in the shield of the cable, current is allowed to leak toward the outside of the cable and generate B_1_. The propagation constant on the inside of the cable does not change depending on antenna loading or bending of the cable. This allows us to produce a relatively flat current distribution on the outside, which reduces peak SAR and improves B_1_ homogeneity. However, the wave impedance on the outside of the cable differs from that on the inside, so internal reflections occur at the gap, where current is brought from the inside to the outside of the cable. Adding inductors to each end of the antenna reduces these internal reflections, essentially ‘matching’ the wave from the inside to the outside. Without inductors, these internal reflections cause a standing wave inside the antenna with high amplitude, which results in high copper losses.

With gaps positioned at 100 mm from the center, an inductor value of 28 nH results in a combination of three beneficial properties: (1) A relatively flat current distribution between the gaps on the outside of the antenna, (2) reduced internal reflections at the gap and (3) an input impedance at the port which can be matched to 50 Ohm using a single parallel capacitor. However, it is also a compromise: (1) the current distribution on the outside is not perfectly flat; a flatter current distribution could be achieved by a lower inductance value. (2) There are still some internal reflections, and the inductor itself carries significant current (roughly 3 times more than the current on the outside of the shield): Total losses on the inductors and inside of the cable are estimated to be around 6% of input power, down from ~11% without the inductors. (3) Variations in antenna loading can severely distort the matching. If loading changes, the internal reflections at the gap change, and the impedance at the source changes. However, the flexible nature of the antenna, combined with firm but flexible foam housing, makes it easy to maintain a 2 cm distance between the antenna and loading tissue. As the antenna follows the shape of the body, loading variations are small and therefore matching remains constant. Note that bending of the antenna itself (under constant loading conditions) does not affect the matching.

With gaps at 125 mm from the center, it is possible to achieve an even flatter current distribution. However, in this case, no inductance value was found that brings the losses inside the antenna down to an acceptable level. Additionally, the gains in terms of SAR efficiency are minimal when compared to the final design with gaps at 100 mm.

The single antenna measurements show 18% less peak heating for the same B_1_. However, these results are not reproduced in the simulations with eight antennas on Duke, where for 1 uT in the heart, the SAR reduction in simulations with eight channels is only 3.6%. A reason for this can be that with a single antenna the highest electric field values are found directly under the antenna and, due to the homogeneous conductivity, this is also where the peak SAR value is located. With an array of eight antennas, on an inhomogeneous human body, the peak SAR is typically caused by the electric fields of multiple antennas interfering constructively somewhere deeper within the body. Therefore, the reduction in peak SAR due to a flatter current on the antenna is expected to be smaller. Additionally, the flexible shape of the coax dipoles allows them to perfectly follow the contours of the body model. Possibly the SAR reduction due to a flatter current distribution is offset by a SAR increase due to antennas on average being closer to the body. Therefore, the main advantage of the coax dipoles is the improved homogeneity. The flexible shape and absence of dielectric spacers also allows the coax dipoles to be positioned closer to each other than the fractionated dipoles, which is what caused the slight increase in coupling. Simulations with two antennas (not shown in this work) have shown that if the distance between two coax or fractionated dipoles is the same, the coupling is the same.

Simulating the fine structures of the coax dipoles with the FDTD method proved to be quite challenging. As the antennas are shaped to follow the contours of the body model, the two arms of some antennas were oriented differently with respect to the rectangular FDTD grid. This resulted in different staircasing patterns on both arms, which initially caused their current distributions to become asymmetric. This was solved by slightly changing the inductance values at the end of each arm, restoring the symmetric current distribution while keeping the real part of the source admittance at 0.02 S.

In vivo, we have demonstrated that the coax dipoles achieve good coverage of the heart by obtaining cardiac cine images. However, due to a combination of breathing motion and blood flow, obtaining reliable B_1_ maps of the heart is notoriously difficult. Therefore, to assess B_1_ efficiency in comparison to the fractionated dipoles, we have chosen the prostate as an imaging target. After the first scanning session, the large variation in B_1_ amplitudes with the fractionated dipoles prompted us to repeat the measurements. We are not entirely certain what causes these variations. Possibly the body curvature prevents the antenna from making good contact with the body over its entire length. Some sections of the antenna were ‘floating’ slightly above the body. This might have changed the amplitude of individual channels. Regarding all available data, we conclude that on average the coax dipoles perform equally well as the fractionated dipoles, but most likely their flexible shape makes their performance more consistent across different subjects.

Accurate assessment of local SAR for the coax dipole on multiple models will be a computationally demanding task, due to the large number of voxels required to accurately model the antenna. However, we estimate that overall the inter‐subject variability of peak local SAR will actually be smaller than with rigid coils. The shape mismatch between body curvature and antenna that exists with rigid coils will be much less with flexible antennas. As a result, local SAR should depend mostly on the electrical properties of the tissue in the target region, and much less on the exact shape. However, this will have to be investigated for each target region.

Improving the SAR efficiency of dipole antennas for high‐field body imaging is an on‐going topic of research, and many have been successful in reducing the SAR caused by the current maximum in the center of the antenna. Zivkovic et al^8^ have presented a *passively fed dipole*, where the dipole antenna is not fed directly by the source, but through coupling via a smaller dipole. Tarakameh et al^32^ have presented a *bumped dipole*, where the feed point of the antenna is moved away from the body. These two solutions both reduce SAR emanating from the feed point. In our design, a similar effect is achieved by having the feed point inside the shield of the coax cable. Steensma et al^5^ have presented a *snake antenna* at 10.5T, which has its geometry optimized for optimal SAR efficiency. In this design, the SAR directly under the feed point in the center is actually slightly lower than under the arms of the antenna. All of these solutions show an increase in SAR efficiency (B_1_/√peak SAR) at the cost of some transmit efficiency (B_1_/√input power). For the coax dipoles the improvement in SAR efficiency is more modest, but it is achieved without sacrificing transmit efficiency. Additionally, the flexible design improves B_1_ uniformity, patient comfort and allows for the dipoles to be used on a wide range of possible imaging targets, such as the carotid arteries, axillary lymph nodes, or shoulder joint.

## 5 CONCLUSIONS

This paper presents the *coax dipole*, a flexible dipole antenna for 7T. Its design is inspired by recent developments of loop antennas made from coaxial cable, and operates based on similar principles. A section of coax cable is driven on the core conductor and, through gaps in the shield, a relatively flat current profile is setup on the outside of the antenna to generate the RF fields. A series of FDTD simulations is used to determine optimal gap positions. Findings show that two lumped inductors at the antenna endings are required to reduce copper losses and facilitate impedance matching. Single antenna measurements indicate the coax dipole produces the same B_1_ as a fractionated dipole with 18% lower SAR. FDTD simulations of an eight‐channel array find a 3.6% reduction in peak SAR and 18% improved B_1_ uniformity in the heart. In‐vivo B_1_ measurements comparing coax dipoles to fractionated dipoles show they perform equally well on average, but the coax dipoles show more consistent performance. The flexible design of the coax dipoles improves patient comfort and allows for the antennas to be used on a wide range of possible imaging targets, without sacrificing performance compared to situations where conventional rigid antennas can be used.

## Supporting information


**VIDEO S1** “four‐chamber view.mp4”: Cardiac cine scan of a healthy volunteer in four‐chamber view obtained using an array of eight coax dipoles

**VIDEO S2** “short‐axis view.mp4”: Cardiac cine scan of a healthy volunteer in short‐axis view obtained using an array of eight coax dipoles

**VIDEO S3** “transverse slice.mp4”: Transverse slice of cardiac cine scan of a healthy volunteer obtained using an array of eight coax dipoles

**VIDEO S4** “two‐chamber view.mp4”: Cardiac cine scan of a healthy volunteer in two‐chamber view obtained using an array of eight coax dipoles
